# Gonadotoxic effect of tramadol administration: A prospective controlled study

**DOI:** 10.1080/2090598X.2021.2002634

**Published:** 2021-11-26

**Authors:** Tarek Soliman, Hussein Shaher, Ahmed Mohey, Waleed El-Shaer, Ahmed Sebaey

**Affiliations:** Department of Urology and Andrology, Benha University, Benha, Egypt

**Keywords:** Tramadol, gonadotoxic, libido, erectile dysfunction

## Abstract

**Objective:**

To detect the possible gonadotoxic effects of tramadol dependence on seminal fluid parameters, and prolactin and testosterone hormone levels.

**Patients, Subjects, and Methods:**

There were 94 participants who were divided into a tramadol-dependent group (T-group; 56 patients) and a control group (C-group; 38 healthy volunteers). The following variables were evaluated: testosterone level, prolactin level, erectile function, libido, semen parameters, and effect of tramadol dose and dependence duration.

**Results:**

There was a significant increase in erectile dysfunction (ED) and decreased libido in the T-group vs C-group. Also, the serum testosterone level was lower in the T-group vs the C-group, while the serum prolactin level was significantly higher in the T-group vs the C-group. All semen parameters were low in the T-group except for abnormal forms, which were high. As the dose of tramadol increased there was a more negative effect on the previous parameter, while ED, libido, semen volume and concentration showed no significant changes. When comparing tramadol doses of 400–1000 mg/day to >1000 mg/day, the tramadol blood level increased with high doses, while serum testosterone level decreased when the dose increased and the prolactin level increased when the dose increased. Progressive motility of the sperm decreased and abnormal forms increased. Also increased duration of tramadol administration was also accompanied by a more negative effect on these parameters

**Conclusion:**

Tramadol administration has a negative effect on hormone levels, libido, erectile function, and semen characters.**Abbreviations:** ED: erectile dysfunction; EF: erectile function

## Introduction

Tramadol is an atypical synthetic opioid and an analgesic. Tramadol has two mechanisms of action through μ-opioid agonistic activity, also gamma-aminobutyric acid (GABA), noradrenaline, serotonergic actions it has a central action and local action through increasing nitric oxide levels [1–3]; so, it is prescribed as pain reliever in moderate and severe cases [4].

Due to its popularity and huge use especially among youth as pain reliever, tramadol dependence has become a problem in the Egyptian community [5,6]. Also a lot of people in Egypt use tramadol as a treatment for premature ejaculation, as they believe that tramadol has a positive impact on their sexual functions [7].

Much research has found that tramadol can produce changes in gonadal hormone levels and increase DNA damage in sperm, which affects spermatogenesis in laboratory animals such as rats [8]. Administration of opioid compounds at high doses and for long periods increases reactive oxygen species, which can lead to DNA fragmentation of sperm cells and affecting sperm motility and morphology. So, it may lead to male infertility [9,10].

Opioids and tramadol as an opioid may have negative effects on libido, and erectile function (EF) and ejaculatory function [11,12]. Long-term use of opioids may result in hypogonadism by decreasing the release of GnRH, so decreasing the testosterone level and finally affecting EF and male fertility [11,12].

The published data about the impact of chronic tramadol addiction on male sexual and reproductive functions are scarce. Thus, we opted to design the present study to investigate the effect of tramadol addiction on male sexual life and fertility.

## Patients, subjects, and methods

This was prospective controlled non-randomised study that enrolled 94 participants who were divided into 56 patients who were tramadol-dependent (T-group) and 38 healthy volunteers, i.e. control group (C-group). These participants were among the attendants of the outpatient clinics, Benha University Hospital, Egypt, in the period between June 2018 and June 2019.

The sample size was calculated with the formulas of a two-sample test. Sampling ratio was 2:3 (N2/N1), the power was 80%, 5% α error and CI of 95%; Calculated minimum sample size was 54 and 36 for the T- and C-groups, respectively.

All participants in the study were informed about the details of the study. And a written, well-informed consent was signed by each participant. The study protocol was approved by the local Ethics Committee.

### Inclusion criteria

**T-group**: adult males aged <40 years, dependent on tramadol only, with fulfilment of the Diagnostic and Statistical Manual of Mental Disorders, fifth edition (DSM V) criteria of dependence [13].

All patients had no objections or medical diseases interfering with obtaining blood and semen samples from them.

Criteria of opioid dependence:

1.Taking the opioid in larger amounts and for longer than intended.
2.Wanting to cut down or quit but not being able to do it.
3.Spending a lot of time obtaining the opioid.
4.Craving or a strong desire to use opioids.
5.Repeatedly unable to carry out major obligations at work, school, or home due to opioid use.
6.Continued use despite persistent or recurring social or interpersonal problems caused or made worse by opioid use.
7.Stopping or reducing important social, occupational, or recreational activities due to opioid use.
8.Recurrent use of opioids in physically hazardous situations.
9.Consistent use of opioids despite acknowledgment of persistent or recurrent physical or psychological difficulties from using opioids.
10.Tolerance as defined by either a need for markedly increased amounts to achieve intoxication or desired effect or markedly diminished effect with continued use of the same amount (does not apply for diminished effect when used appropriately under medical supervision).
11.Withdrawal manifestation.
  - The presence of two to three symptoms out of the 11 is defined as mild.
  - The presence of four to five symptoms is defined as moderate.
  - The presence of six or more symptoms is defined as severe

**C-group**: healthy adult males of the same age range with normal libido, EF, normal hormonal levels, and normal semen parameters.

### Exclusion criteria

Patients with chronic disease such as: diabetes, obesity (body mass index ≥30 kg/m^2^), bronchial asthma, hepatic, renal, cardiovascular, or skin diseases, cancer, and those under radio- or chemotherapy. Also, patients dependent on mixed drugs or under hormonal treatment or on phosphodiesterase type 5 inhibitors were excluded. Moreover, patients with varicocele or testicular tumours that may affect fertility status, patients with infertility were excluded.

All participants were subjected to history taking including toxicological history as concentration, amount and form of tramadol received per day. Duration of tramadol dependence (lifetime use of tramadol). Any previous treatment for dependence. Presence or absence of libido and EF were also evaluated.

### Investigations

#### Quick view tramadol test card

A one-step drug of abuse rapid test: it is an *in vitro* immuno-chromatographic assay for the rapid visual qualitative detection of tramadol in human urine to identify positive cases who then had for tramadol their blood level investigated.

#### Semen analysis

Participants were asked to give the sample in the laboratory by masturbation after avoiding ejaculation for 72 h; and avoiding alcohol, caffeine, and drugs such as cocaine and marijuana for 5 days before the test. Also, patients were asked to stop any herbal or hormonal medications. Assessment of semen by using computer-assisted semen analysis (CASA) and according to World Health Organization reference values for human semen characteristics (2010) for semen volume (mL), concentration (million/mL), motility (total and progressive motility) and abnormal forms [14].

#### Testosterone and prolactin levels

Due to the fluctuation of testosterone levels throughout the day blood samples were taken between 07:00 hours and 10:00 hours to measure total serum testosterone and prolactin level (ng/dL) by Elecsys Testosterone II and Elecsys Prolactin II (Roche Diagnostics, Mannheim, Germany).

#### Erectile function

The EF of all participants were evaluated by the validated Arabic version of the Sexual Health Inventory for Men (SHIM), which contains five questions, each of them scoring from 1 to 5. A final score of 22–25 indicated no erectile dysfunction (ED), 17–21 mild ED, 12–16 mild-to-moderate ED, 8–11 moderate ED, and 1–7 severe ED [15].

#### Scrotal duplex ultrasonography

Scrotal duplex ultrasonography was used to exclude varicocele and any other pathology affecting testicular function.

In this study we classified the T-group according to dose: 400–1000 mg/day (Group 1) and >1000 mg/day (Group 2). We also we classified the T-group according to duration of addiction to: Group (A): duration 1–4 years (Group A) and >4 years (Group B).

Data were expressed as a mean (standard deviation [SD]) and were statistically analysed using the Statistical Package for the Social Sciences (SPSS®), version 20 (IBM corp., Armonk, NY, USA). The independent Student’s *t*-test and chi-square test were applied when appropriate. A *P* < 0.05 was considered to indicate statistical significance.

## Results

The demographic data of the participants are detailed in Table 1, regarding age there was no significant difference between The T-group and the C-group, while for education there was a significant difference between the T-group and C-group.

**Table 1.** Comparison between the T-group and C-group according to age, education, hormone levels, and seminal parameters

Variable	T-group (*N* = 56)	C-group (*N* = 38)	St *t*-test	*P*
Age, years, mean (SD)	28.52 (4.18)	28.84 (4.27)	0.37	0.72
Education, *n* (%) Not educated Educated	39(69.6) 17(30.4)	8(21.1) 30(78.9)	χ^2^ = 21.38	<0.001*
ED, *n* (%) Yes No	23(41.1) 33(58.9)	3(7.9) 35(92.1)	χ^2^ = 12.45	<0.001*
Libido, *n* (%) Decrease Normal	26(46.4) 30(53.6)	5(13.2) 33(86.8)	χ^2^ = 11.34	0.001*
Serum testosterone level, ng/dL, mean (SD)	392.32(118.06)	688.29(210.43)	8.71	<0.001*
Serum prolactin level, ng/mL, mean (SD)	22.11 (3.89)	8.74 (2.01)	19.48	<0.001*
Semen volume, mL, mean (SD)	1.49 (0.29)	2.04 (0.24)	9.56	<0.001*
Semen concentration, million/mL, mean (SD)	14.27 (1.98)	30.84 (2.66)	34.59	<0.001*
Semen total motility, %, mean (SD)	32.2 (4.08)	52.23 (3.95)	23.68	<0.001*
Progressive motility, %, mean (SD)	22.83 (3.53)	39.98 (3.27)	23.82	<0.001*
Abnormal forms, %, mean (SD)	52.29 (9.13)	28.37 (4.69)	14.85	<0.001*

*Significant at *P* < 0.05.

There was a significant difference was in ED and decreased libido, which occurred respectively in 41.1% and 46.4% in the T-group and in 7.9% and 13.2% in the C-group (Table 1).

Also, there was a statistically significant decrease in the serum testosterone level and a significant increase in the serum prolactin level in the T-group compared to the C-group (Table 1).

There was a significant decrease in seminal fluid volume, sperm concentration, total motility, and progressive motility in the T-group compared to the C-group. While abnormal forms of sperm were significantly increased in the T-group compared to the C-group (Table 1).

Regarding the dose of tramadol, when the tramadol dose was increased there was a significant increase in ED and decrease in libido when comparing in Group 1 and Group 2 (Table 2). Regarding the duration of intake, there was significant increase in ED, which was 8.7% in Group A vs 63.6% in Group B, and significant decrease in libido, which was 13% in Group A and 69.7% in Group B (Table 3).

**Table 2.** Comparison between effects of tramadol dose on ED, libido, hormone levels, and semen parameters

T-group (*N* = 56)	Group 1 Dose 400–1000 mg/day (*n* = 36)	Group 2 Dose >1000 mg/day (*n* = 20)	St *t*-test	*P*
Tramadol blood level, mg, mean (SD)	415.97 (86.96)	745.95 (54.07)	15.37	<0.001*
ED, *n* (%) Yes No	5 (13.9) 31 (86.1)	18 (90.0) 2 (10.0)	χ^2^ = 30.77	<0.001*
Libido, *n* (%) Decrease Normal	6 (16.7) 30 (83.3)	20 (100) 0 (0.0)	χ^2^ = 35.9	<0.001*
Serum testosterone, ng/dL, mean (SD)	451.39 (106.48)	286.0 (30.85)	6.77	<0.001*
Serum prolactin, ng/mL, mean (SD)	19.69 (2.36)	26.45 (1.61)	11.38	<0.001*
Semen volume, mL, mean (SD)	1.51 (0.27)	1.45 (0.33)	0.71	0.48
Semen concentration, million/mL, mean (SD)	14.43 (2.01)	13.99 (1.94)	0.81	0.42
Semen total motility, %, mean (SD)	34.53 (2.99)	28.0 (1.68)	8.98	<0.001*
Semen progressive motility, %, mean (SD)	24.9 (2.48)	19.09 (1.36)	9.66	<0.001*
Abnormal forms, %, mean (SD)	46.17 (4.03)	63.3 (3.6)	15.81	<0.001*

*Significant at *P* < 0.05.

**Table 3.** Comparison between effects of tramadol addiction duration on ED, libido, hormone levels, and semen parameters

T-group (*N* = 56)	Group A Duration 1–4 years (*n* = 23)	Group B Duration >4 years (*n* = 33)	St *t*-test	*P*
Tramadol blood level, mg, mean (SD)	357.52 (41.71)	656.7 (121.04)	11.37	<0.001*
ED, *n* (%) Yes No	2 (8.7) 21 (91.3)	21 (63.6) 12 (36.4)	χ^2^ = 16.9	<0.001*
Libido, *n* (%) Decrease Normal	3 (13.0) 20 (87.0)	23 (69.7) 10 (30.3)	χ^2^ = 17.49	<0.001
Serum testosterone, ng/dL, mean (SD)	517.83 (69.15)	304.85 (38.17)	14.79	<0.001*
Serum prolactin, ng/mL, mean (SD)	18.17 (1.4)	24.85 (2.4)	11.97	<0.001*
Semen volume, mL, mean (SD)	1.79 (0.16)	1.28 (0.16)	11.82	<0.001*
Semen concentration, million/mL, mean (SD)	15.83 (0.59)	13.18 (1.87)	6.55	<0.001*
Semen total motility, %, mean (SD)	36.43 (1.42)	29.24 (2.3)	13.31	<0.001*
Semen progressive motility, %, mean (SD)	26.57 (1.24)	20.22 (1.8)	14.64	<0.001*
Abnormal forms, %, mean (SD)	43.91 (3.23)	58.12 (7.12)	8.93	<0.001*

*Significant at *P* < 0.05.

There was a significant decrease in the serum testosterone level in Group 1 and in Group 2, and a significant increase in the serum prolactin level occurred in Group 1 and in Group 2 when the tramadol dose was increased (Table 2). There was a significant decrease in the testosterone level in Group A and Group B accompanied by a significant increase in the prolactin level in Group A and Group B when the duration of administration was increased (Table 3).

There was no significant relation between tramadol dose and both the volume of semen and the concentration of sperm either in Group 1 or Group 2. In contrast, total and progressive motility significantly decreased as the dose of tramadol increased in Group 1 and in Group 2. However, increasing the dose of tramadol resulted in a significant increase in the abnormal forms of sperm in Group 1 and in Group 2 (Table 2). The semen volume, sperm concentration, total and progressive motility revealed significant decrease when the duration of dependence increased as shown in groups A and B, while the abnormal forms showed a significant increase when the duration of administration was prolonged (Table 3).

## Discussion

The wide availability of tramadol as a pain killer for many forms of pain either acute or chronic could be a main factor for its widespread use and abuse [16,17]. Another factor for the widespread use of tramadol is its role in treatment of premature ejaculation, which may be considered one of the apparent causes of increased magnitude of the problem among youth who believe that tramadol can improve their sexual performance [16,17].

Our present study showed the effects of tramadol dependence on EF, libido, seminal fluid parameters as well as testosterone and prolactin hormonal levels. It was carried on 94 subjects, 56 in the T-group and 38 in the C-group.

In the present study the selected age of the participants was <40 years to exclude the ageing effect on semen parameters and hormonal levels, as Eskenazi et al. [18] reported significant age-related decreases in semen quality mostly for semen volume and sperm motility, and they suggested that men may became progressively less fertile as they aged.

Concerning the relationship between tramadol dependence and age in the present study, the mean (SD) age of the T-group was 28.52 (4.18) years. This is in agreement with Shadnia et al. [19], Iravani et al. [20], and Zabihi et al. [21] who stated that the age group of 20–45 years was the highest for drug and substance abuse compared with other age groups.

There was a significant difference between tramadol-dependent patients and the controls in educational status, as 69.6% in the T-group were uneducated compared with ~21.1% in the C-group. This finding agreed with Fawzui [22] who stated that most tramadol dependents in the Ain Shams Toxicology Unit were uneducated or had a minimal educational level.

In the present study, the majority the patients (36) in the T-group received tramadol in a dosage range from 400 to 1000 mg/day, with 20 subjects having a dosage of >1000 mg/day. This concurs with the Goda [23] study of 100 tramadol-dependent patients, as he reported that 24% of his patients’ received tramadol in dosage of <400 mg/day, 53% of them received it in a dosage range of 400–2000 mg/day, and 23% of them received >2000 mg/day.

As regard the duration of tramadol dependence, 23 patients in the T-group used tramadol for 1–4 years and 33 had used it for >4 years. Comparable results were obtained by Goda [23], as 20% of his cases used tramadol for <2 years, 44% of them used it for 2–7 years, and 36% of them used it for >7 years.

There were significant differences in the serum testosterone and prolactin levels between the T-group and C-group in the present study. This is compatible with the study done by Daniell [24], who measured the hormonal profile in 54 community-dwelling outpatient men consuming oral forms of opioids several times daily, and he reported significant decreased levels of serum testosterone with increased prolactin levels, referring this to the disturbance of hypothalamic–pituitary–gonadal axis. Testosterone was also reduced due to direct inhibition of testicular testosterone synthesis [24].

Auernhammer et al. [25] stated that opioids bind to specific receptors in the hypothalamus and pituitary gland, interfering with production of corticotrophin-releasing hormone (CRH) and adrenocorticotrophic hormone (ACTH), and an obstacle to the release of cortisol and androgen precursors. Chan et al. [26] also suggested that the lowering of the serum testosterone level by tramadol might result from adrenal insufficiency secondary to chronic use.

For the seminal fluid parameters, there were significant differences in semen volume, sperm concentration, motility, and abnormal forms between the T-group and C-group in our present study. This is in agreement with Ragni et al. [27] who recorded significant teratozoospermia, oligospermia and diminished sperm motility in their dependent cases. Furthermore, Ragni et al. [27] revealed abnormal semen parameters in cases of prolonged opioid administration in the form of asthenospermia (100% of cases), teratozoospermia and hypospermia (24%), oligospermia (17%), and they concluded that seminal pathology even with normal hormone levels might be an early indication of opioid-induced gonadal dysfunction.

In the present study, a significant difference was detected regarding EF and decreased libido when comparing the T-group and C-group. This is consistent with other studies that reported hypogonadism as a serious impact of long-term tramadol administration. Symptoms of tramadol-induced hypogonadism include loss of libido, infertility, fatigue, depression, anxiety, loss of muscle strength and ED in men [24,27].

Daniell [24] contributed the ED and decreased libido to the subnormal sex hormone levels induced by chronic tramadol administration that predispose to a diminished quality of sexual life in his studied cases. Furthermore, Katz et al. [28] reported that long-term tramadol administration often induces hypogonadism. This is in contrast to the results of El-Hadidy and El-Gilany [29] who evaluated 112 tramadol-dependent married men. They noted that the subscales of sexual relationship, sexual self-esteem, and overall sexual satisfaction showed a significant increase after treatment compared with before treatment. This could be explained by the fact that patients on tramadol knew that they were abnormal and dependent on the substance to allow efficient sex practice. The explanation of this difference could be due to the use of tramadol on demand to treat premature ejaculation but not tramadol dependence with regular use for >1 year [29].

There was a significant increase in tramadol blood levels as the daily received dose was increased in the present study. Similar finding observed by Deer and Gunn [30] who also reported that measuring tramadol blood level provides extremely valuable information, which can be used to evaluate impairment or overdose cases. Furthermore, Cepeda et al. [31] stated that elevated or diminished tramadol blood levels could guide the clinician to select the dose and prevent overdose or underdose (withdrawal).

In the present study, it was noticed that as the dose and duration of tramadol increased the testosterone level decreased and prolactin level increased. This agrees with the result of McKim [32] who reported that changes in sex hormones levels are dependent on the administered dose of tramadol.

Gowing et al. [33] and Herzog et al. [34] found that the influence of tramadol on testosterone and prolactin serum levels is significantly related to its dose. Large doses of tramadol administration led to more effects. Moreover, an animal study by El-Gaafarawi [4] showed minor changes in testosterone and prolactin levels in rats that received 40 mg/kg tramadol after 30 days, but 80 mg/kg tramadol exerted moderate effects at 20 and 30 days. Additionally, Yilmaz et al. [35] reported that chronic opioid exposure may cause long-term endocrine disturbances in rats subcutaneously injected with opioid (5 mg/kg) twice daily for 30 days, with further reduction in serum testosterone as the duration of exposure prolonged. McKim [32] also stated that tramadol is known to decrease the levels of sex hormones and this lowered hormonal level is thought to be a chronic outcome.

In the present study, total and progressive sperm motility significantly decreased as the dose of tramadol increased. Also, increasing the tramadol dose resulted in an increase in abnormal forms of sperm. This is in accordance with El-Ghawet [36] who stated that tramadol produced a concentration-dependent effect on sperm motility and abnormal forms, linking this to more cellular damage and the more oxidative stress caused by higher doses of tramadol. Furthermore, Omid et al. [37] found a positive correlation between the dose and abnormal semen parameters in mice, indicating that the effect of tramadol on semen quality is dose dependent.

There was a significant relationship between the dose on both ED and decreased libido in the present study. Daniell [24] reported that ED and decreased libido had a dose-related pattern in his study. Additionally, Goodyear-Smith et al. [38] stated that reduced libido and ED were dose-dependent effects, and both were improved by lowering the administered dose.

The present study revealed a significant positive relationship between the duration of tramadol dependence and tramadol blood level. Similarly, Fudin [39] found a linear relationship between blood tramadol level and the period of exposure and stated that a duration-response can be predicted in patients with chronic tramadol toxicity.

There was a significant negative correlation between the tramadol blood level and serum testosterone level, while the opposite was the case for the prolactin level in the T-group in the present study. In line with this result, Deer and Gunn [30] reported low testosterone level and high prolactin level with increasing tramadol blood level among cases of chronic tramadol administration. They also recommend measuring serum testosterone level in patients on long-term therapy with tramadol to avoid hypogonadism.

In the present study, for the relationship between the duration of tramadol dependence and the abnormal semen parameters it was found that seminal volume, sperm concentration, and total and progressive motility had a significant negative relation. Conversely, abnormal forms of sperms had a significant positive relation with the duration of dependence.

Ahmed and Kurkar [40] stated that decreased semen quality was noted in chronic tramadol administration. This could be due to the more degenerative changes of the testis with the more prolonged duration of tramadol use.

In our present study, increased duration of dependence led to a significant increase in both ED and decreased libido. This is in agreement with Daniell [24] who stated that sexual dysfunction from tramadol should be kept in mind during long-term therapy, especially when in large doses. Additionally El-Ghawet [36] concluded that chronic tramadol administration led to reproductive dysfunction and increased the likelihood of infertility.

There were limitations to the present study as there was a small number of participants and it was a non-randomised study, thus a larger multicentric study is needed. Also, follow-up after cessation of tramadol and other opioids agents needs to be studied.

## Conclusion

Tramadol administration has a negative effect on libido, EF, hormone levels (testosterone and prolactin), and semen characteristics.
